# Prognostic Values and Underlying Regulatory Network of Cohesin Subunits in Esophageal Carcinoma

**DOI:** 10.7150/jca.66949

**Published:** 2022-03-06

**Authors:** Wenqiang Gan, Weiqi Wang, Tiegang Li, Rixin Zhang, Yufang Hou, Silin Lv, Zifan Zeng, Zheng Yan, Min Yang

**Affiliations:** State Key Laboratory of Bioactive Substances and Function of Natural Medicine, Institute of Materia Medica, Chinese Academy of Medical Sciences and Peking Union Medical College, Beijing 100050, China.

**Keywords:** STAG2, RAD21, esophageal carcinoma (ESCA), biomarker, prognosis

## Abstract

**Background:** Cohesin is a highly conserved and ubiquitously expressed protein complex. While increasing evidence suggests that cohesin dysregulation is vital in the carcinogenesis of numerous malignancies, little is known about the prognostic values and potential mechanisms of cohesin subunits and direct regulators in esophageal carcinoma (ESCA).

**Methods:** RNA-sequencing data from The Cancer Genome Atlas (TCGA) and Genome Tissue Expression (GTEx) were used. The subunits and regulators of cohesin affecting the prognosis of ESCA were screened by Kaplan-Meier survival analysis; univariate and multivariate Cox regression analyses were performed; and the receiver-operating characteristic (ROC) curve was determined. The ESCA hazard model and nomogram map were constructed by integrating the clinical data. We used functional analysis and protein-protein interaction (PPI) networks to explore underlying pathways. Finally, immunohistochemistry was performed to examine the expression levels of cohesin subunits in tissue microarray (TMA).

**Results:** Transcriptome data from multiple ESCA patient datasets showed cohesin subunits SMC1A, SMC1B, SMC3, STAG1, STAG2, RAD21, and cohesin regulators including ESCO2, NIPBL, MAU2, WAPL, PDS5A and PDS5B were all upregulated in ESCA tissues compared to normal tissues. Survival analysis demonstrated that high STAG2 expression was significantly associated with poorer overall survival (OS) and progression-free survival (PFS) in esophageal adenocarcinoma (EAC). In contrast, high RAD21 expression was significantly correlated with better OS in esophageal squamous cell carcinoma (ESCC). Moreover, STAG2 and RAD21 were identified as independent prognostic factors and predictive biomarkers in EAC and ESCC, respectively. Functional enrichment analysis further revealed that STAG2 and RAD21 were mainly involved in the mitotic nuclear division, DNA repair, angiogenesis, epithelial-mesenchymal transition (EMT), and oncogenic signaling pathways. PPI analysis illustrated that STAG2 and RAD21 could cross-talk through cancer-associated modules and performed the core roles of the whole PPI network. Using TMA, STAG2 protein expression positively correlated with lymph node metastasis and advanced clinical stage of EAC patients, whereas there was a negative correlation between RAD21 protein expression and the malignant clinicopathological parameters in ESCC.

**Conclusion:** These findings suggest that STAG2 and RAD21 can be used as predictive biomarkers for risk assessment and prognostic stratification in ESCA, which provide potential novel insights into molecular targets of ESCA.

## Introduction

Esophageal carcinoma (ESCA) was the 14^th^ most common cancer reported in 2020, ranking eighth in incidence and sixth in all cancer-related deaths worldwide 1. The majority of esophageal cancers can be histologically divided into two subtypes, esophageal squamous cell carcinoma (ESCC) and esophageal adenocarcinoma (EAC), with dysplasia in the squamous epithelium and precursor lesions of Barrett's esophagus, respectively 2. For ESCA, the prognosis as yet mainly depends on the clinical stage, and the survival rate per stage is similar between EAC and ESCC 3. However, ESCC is different from EAC in etiology, epidemiology, and pathophysiology 3, 4. Molecular characteristics also reveal distinct differences between ESCC and EAC 5. Furthermore, ESCC is more sensitive to chemoradiation than EAC in clinical treatment 6.

Cohesin is one of the eukaryotic structural maintenance of chromosome (SMC) complexes and is a large ring-shaped protein complex composed of the core subunits SMC1 (structural maintenance of chromosomes 1), SMC3 (structural maintenance of chromosomes 3), STAG (stromal antigen, either STAG1 or STAG2), and RAD21 (RAD21 cohesin complex component) 7, 8. The main functions of this complex are central in regulating sister chromatid cohesion, chromosome organization, gene expression, and DNA repair 9. Germline mutations in primary genes associated with the cohesin complex can cause human developmental disorders such as Cornelia de Lange syndrome and Roberts syndrome 10, 11. Moreover, genetic alterations in genes encoding cohesin subunits have been identified in bladder cancer, melanoma, myeloid malignancies, colorectal cancer, and lung cancer 12-15. Expression levels of cohesin component genes are associated with tumor prognosis and metastatic behavior. For example, high expression of SMC1A in colorectal cancer can promote tumor development 16, 17. Overexpression of SMC3 in human colon carcinoma and mouse intestinal tumor can trigger cell phenotypic transformation 18, 19. In bladder cancer, STAG2 loss of expression is associated with recurrence and disease prognosis 20. Moreover, overexpression of RAD21 in sporadic epithelial breast cancer and in a subset of familial breast cancer was associated with disease progression, poor disease outcome, or chemotherapeutic resistance 21, 22. At the same time, low RAD21 expression may directly cause apoptosis resistance in oral carcinoma cells 23. However, the prognostic values of cohesin subunits in ESCA have rarely been reported.

The present study evaluated the gene expression levels of cohesin subunits and regulators in normal individuals and ESCA patients based on the Genotype-Tissue Expression (GTEx) and The Cancer Genome Atlas (TCGA) database. The underlying biological functions, relevant pathways, and protein-protein interactions of cohesin subunits in ESCA were also explored via comprehensive bioinformatic analyses. Furthermore, the protein levels in ESCA samples were verified using immunohistochemical (IHC) staining. We revealed the prominent roles of cohesin subunits in the risk assessment and prognostic stratification in ESCA and provided potential novel insights into further investigation of ESCA.

## Methods

### Data Resource

RNA-sequencing data for TCGA ESCA and healthy human tissues were downloaded from the TCGA-TARGET-GTEx cohort using the University of California Santa Cruz (UCSC) Xena browser (https://xenabrowser.net) 24. mRNA expression data of 181 ESCA samples and 651 normal tissue samples in transcripts-per-million format and the matched clinical characteristics of patients were obtained from the cohort to perform subsequent analysis. The transcript count format of mRNA expression data of TCGA ESCA was downloaded from Genomic Data Commons portal (https://portal.gdc.cancer.gov/) to perform differentially expressed genes analysis. The mRNA expression data from various cancer types were acquired from the Oncomine (https://www.oncomine.org/resource) database 25. The *P*-value threshold was 0.05, the fold-change threshold was all, the gene-rank threshold was all, and the data type was mRNA. Methylation analysis of cohesin subunit genes in TCGA ESCA samples was performed using the interactive University of Alabama Cancer Database (UALCAN, http://ualcan.path.uab.edu/analysis.html) web server 26. Genetic alteration analysis of cohesin subunit genes in TCGA ESCA samples was carried out using the interactive cBioPortal (http://www.cbioportal.org/study) web server 27, 28.

### Survival analysis

To adapt the data to the survival analysis, we used the median values of cohesin subunits and direct regulators mRNA expression as the cutoff point to divide the patients into two groups of equal size. This method was also adopted in subtype analyses. Kaplan-Meier curves were used to compare the overall survival (OS) and progression-free survival (PFS) between the two groups using the survfit function in the R package “survminer.” A Log-rank test was performed to estimate the differences between survival statuses. Univariate and multivariate analyses of the Cox proportional hazards regression models were conducted to estimate the hazard ratio with 95% confidence intervals and statistical significance. The results were illustrated using a forest plot via R package “ggplot2.” The prognostic values of STAG2 and RAD21 expression in EAC and ESCC, respectively, were assessed with the median cutoff value using a nomogram via R package “rms.”

### Receiver-operating characteristic (ROC) analysis

ROC analysis was carried out to assess the diagnostic accuracy for OS, five-year survival, and area under the curve (AUC), and the *P*-value was calculated using the Predictive Analytics Software (PASW) Statistics version 18.0 software program (IBM Corporation, Armonk, NY, USA).

### Identification of DEGs

Patients were classified into two groups (low and high STAG2 expression in EAC or low and high RAD21 expression in ESCC) across TCGA datasets. Linear models were used to identify differentially expressed genes (DEGs) between these two groups using the R package “limma.” A false-discovery rate (FDR) adjusted *P*-value of less than 0.05 combined with a simultaneously absolute value of log2 (fold change) of at least 0.58 was set as the threshold for DEG identification. The DEGs selected were visualized by volcano plots and heat maps using the R packages “gghplot2” and “pheatmap.”

### Gene Ontology (GO) and Kyoto Encyclopedia of Genes and Genomes (KEGG) pathway enrichment analyses of the DEGs

GO and KEGG enrichment analyses of the DEGs identified were conducted using the R package “clusterProfiler.” Biological processes (BP), molecular function (MF), and cellular components (CC) were uncovered in the GO enrichment analysis. Only terms with an FDR adjusted *P*-value of less than 0.05 were deemed statistically enriched. The top 10 enriched terms ordered by an ascending *q*-value (a statistical value for estimating false discovery rate) are shown in the bubble chart.

### Gene set enrichment analysis (GSEA) of the DEGs

GSEA (version 4.1.0) was performed to evaluate the correlations in STAG2 expression (high vs. low) and RAD21 expression (high vs. low) using the TCGA dataset. The annotated gene set (h.all.v7.4.symbols.gmt) was used as the reference gene set. To determine the enriched pathways, the number of permutations was set at 1000. Then, the normalized enrichment score and FDR-adjusted *P*-value were measured to indicate significantly enriched gene sets and pathways.

### Protein-protein interaction (PPI) network construction

A PPI network involving 59 proteins was constructed and analyzed with the online Search Tool for the Retrieval of Interacting Genes (STRING) database (https://string-db.org/), followed by reconstruction with the Cytoscape software (version 3.8.0, https://cytoscape.org/) after removal of the isolated nodes. The minimum required interaction score was 0.400. The protein molecules were separated into the following six groups based on their sources from the Database for Annotation, Visualization and Integrated Discovery (DAVID) database: (I) the proteins exhibited in the transforming growth factor beta (TGF-β) signaling pathway; (II) the proteins exhibited in the cell cycle pathway; (III) the proteins exhibited in the pluripotency of stem cells pathway; (IV) the proteins exhibited in the pathway in cancer; (V) the proteins exhibited in adenocarcinoma disease; and (VI) the proteins exhibited in EAC disease.

### Immunohistochemical (IHC) staining of tissue microarray (TMA)

An esophagus cancer tissue array (containing tissue from 35 cases of adenocarcinoma and five normal tissue samples, duplicated cores per case; #DES8011a) and an esophagus squamous cell carcinoma tissue microarray (containing 68 cases of squamous cell carcinoma, two of adenocarcinoma, two of esophagitis, and three samples of normal esophagus tissues, duplicate cores per case; #DES1502) were obtained from Taibosi Biotechnology Co., Ltd. (Xi'an, China). The diagnosis was based on histology. In addition, the clinicopathological information, including age, gender, grade, T stage, lymphatic metastasis, distant metastasis, pathologic stage for all patients, was obtained. This retrospective study using a commercial TMA was performed for scientific research purposes only. The patient-sensitive clinical information was kept anonymous.

For IHC staining, the TMA specimens were deparaffinized, hydrated, and incubated with 3% H_2_O_2_ (349887, Fluka™ Honeywell, USA) for 10 min to quench endogenous peroxidase activity. We then boiled the samples with citrate buffer (pH 6.0, P0081, Beyotime, China) for 90 sec in a steamer for antigen retrieval. The specimens were then blocked with 5% bovine serum albumin for 30 min and incubated overnight with the sheep anti-human primary antibody (anti-STAG2 antibody, #HPA002857, Sigma-Aldrich, Germany; anti-RAD21 antibody, #ab217678, Abcam, UK) at 4°C. We then incubated the specimens with a goat anti-rabbit horseradish peroxidase-conjugated secondary antibody (#305-035-003, Jackson ImmunoResearch, USA) for 30 min at 37°C. After washing, the specimens were then incubated with 3, 3'-diaminobenzidine and counter-stained with hematoxylin.

The slides were digitally analyzed and evaluated using an Aperio ScanScope (Leica Biosystems, Wetzlar, Germany) with the positive pixel counting algorithm, which scored the staining as negative, weak-positive, medium, or strong. The histological score (HS) for each sample was calculated using the following formula: 1 × (% weak staining) + 2 × (% moderate staining) + 3 × (% strong staining). The values for the HS ranged from 0 to 300. The slides were independently reviewed by two experienced pathologists who were blinded to the clinical parameters.

### Statistical analysis

Data analysis and visualization were performed using R software (version 4.0.0) with appropriate packages and GraphPad Prism 8.0 (GraphPad Software Inc., San Diego, CA, USA). For continuous variables, multiple groups were compared by one-way analysis of variance, whereas the student's *t*-test was used to compare two groups. A two-sided *P*-value of less than 0.05 was considered statistically significant.

## Results

### Expression levels of cohesin subunits and direct regulators are upregulated in ESCA and altered in different cancer types

**Figure 1:**
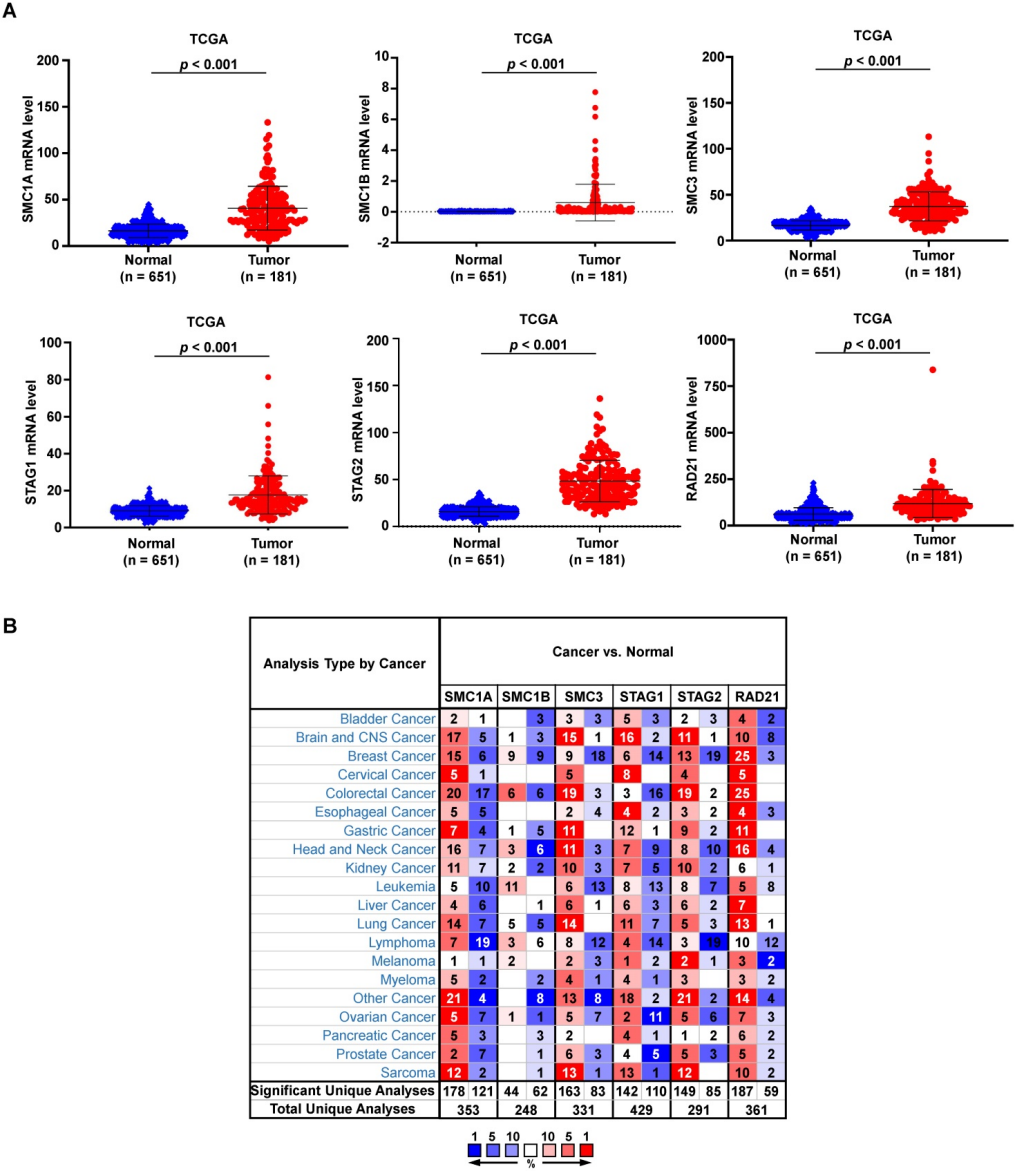
**mRNA levels of cohesin subunits were upregulated in the ESCA tissues and are altered in different human cancers.** (A) Relative mRNA expression levels of cohesin subunits in normal esophageal and ESCA tissues from the GTEx and TCGA-ESCA datasets. (B) Oncomine database analysis results of cohesin subunit mRNA levels in tumor and normal tissues in human cancers. Note: Red and blue denote upregulation and downregulation of the genes of cohesin subunits in the tumor tissues, respectively.

In total, we analyzed the RNA-sequencing data of 651 normal esophagi from GTEx and 181 ESCA tissues from TCGA-ESCA databases to identify the expression feature of cohesin subunit and regulator genes in ESCA patients. The mRNA levels of all cohesin subunits and regulators were significantly upregulated in the ESCA tissues (Figure 1A and Supplementary Figure S1A). However, Oncomine database analysis showed that genes of cohesin subunits and regulators were differentially regulated in esophageal cancer and other cancer types because the expression patterns in different datasets conflicted (Figure 1B and Supplementary Figure S1B).

We performed DNA methylation and gene alteration analyses through the interactive UALCAN and cBioPortal web servers to identify the epigenetic status of cohesin subunit and regulator genes in ESCA patients. Only STAG2 exhibited an obvious lower DNA methylation level in primary ESCA than normal esophageal tissues. While SMC1B, STAG1, and MAU2 sister chromatid cohesion factor (MAU2) showed higher DNA methylation levels in ESCA than normal tissues (Supplementary Figure S2), the other subunit and regulator genes including SMC1A, SMC3, RAD21, establishment of sister chromatid cohesion N-acetyltransferase 2 (ESCO2), nipped-B-like protein (NIPBL), wings apart-like protein homolog (WAPL), PDS5 cohesin-associated factor A (PDS5A), and PDS5 cohesin-associated factor B (PDS5B) showed no significant differences. In total, 3.0%, 1.6%, 1.1%, 9.0%, 3.0%, 10.0%, 2.7%, 13%, 5%, 2.2%, 2.7%, and 7% of the TCGA-ESCA patients showed genetic alterations in the SMC1A, SMC1B, SMC3, STAG1, STAG2, RAD21, ESCO2, NIPBL, MAU2, WAPL, PDS5A, and PDS5B genes, respectively (Supplementary Figure S3A). However, mRNA expression Z-scores relative to diploid samples and Spearman's correlation analysis between copy number alteration (CNA) fraction and mRNA levels showed little relevance between genetic alteration of cohesin subunit and regulator genes and their mRNA expression (Supplementary Figures S3A and S3B).

To evaluate the prognostic value of cohesin subunit and regulator genes, we divided ESCA patients from the TCGA dataset into high and low expression groups according to the median value of each gene expression level. Kaplan-Meier survival curve analysis showed that ESCA patients with high STAG2 or SMC1B expression levels had shorter OS (*P* = 0.020) than those with low STAG2 or SMC1B expression levels, while other groups displayed no significant differences (Supplementary Figures S4A and S4B). To explore the clinical prognostic significance of STAG2 and SMC1B in ESCA, we performed Cox regression analysis. Univariate Cox regression analysis showed that STAG2, SMC1B, M stage, N stage, clinical stage, and residual tumor status were significantly associated with the OS of ESCA patients (*P* < 0.05; Supplementary Figure S5). However, multivariate Cox regression analysis showed that only residual tumor status was an independent prognostic factor for OS in ESCA patients (*P* < 0.05; Supplementary Figure S5).

### High STAG2 expression predicts poor prognosis of EAC patients while high RAD21 predicts a better prognosis of ESCC patients

**Figure 2:**
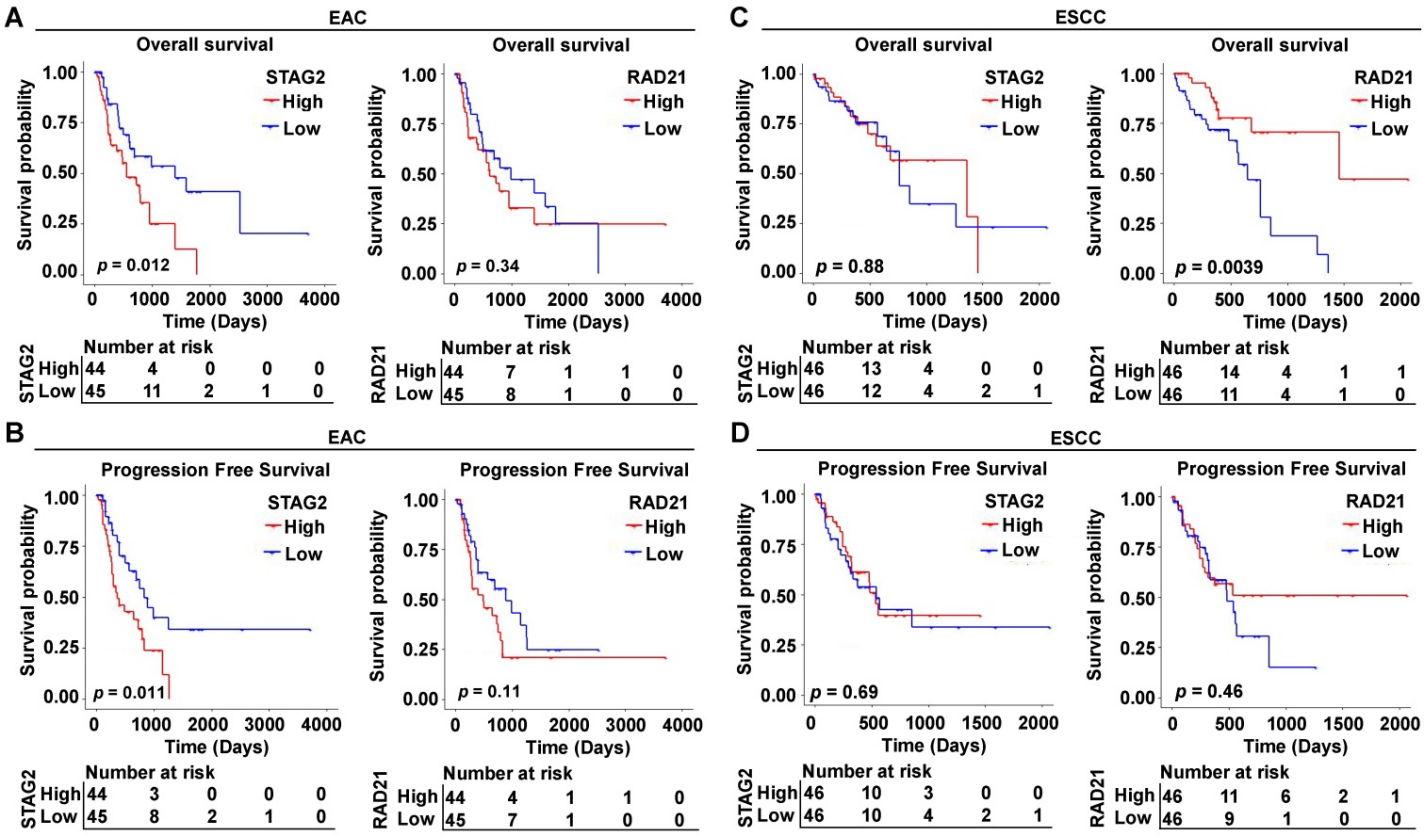
** High STAG2 expression correlated with poor survival outcomes in EAC patients, while high RAD21 expression correlated with better survival outcomes in ESCC patients.** Kaplan-Meier survival curves show **(A)** OS and **(B)** PFS of the EAC patients and **(C)** OS and **(D)** PFS of the ESCC patients with high- and low-expression of STAG2 and RAD21 from the TCGA database.

To explore the prognostic function of cohesin subunits in different histological classifications of ESCA patients, we stratified the 181 TCGA-ESCA patients into 89 EAC and 92 ESCC patient subtypes according to their histological type in clinical data. The median expression value of each subunit gene was used to divide the subtype group patients into high- and low-expressed groups. Survival analysis and Cox regression analysis were performed in each subtype to evaluate the prognostic value of the four subunit genes. In Kaplan-Meier survival curve analysis, EAC patients with high STAG2 expression had shorter OS (*P* = 0.012) (Figure 2A) and PFS (*P* = 0.011) than those with low STAG2 expression (Figure 2B). In contrast, ESCC patients with high RAD21 expression had longer OS (*P* = 0.0039) (Figure 2C) and PFS (*P* = 0.46) tendencies than those with low RAD21 expression (Figure 2D). However, neither EAC nor ESCC patients with high SMC1A, SMC1B, SMC3, or STAG1 expression levels showed significant differences in OS or PFS compared to those with low gene expression levels (Figure S6).

**Figure 3:**
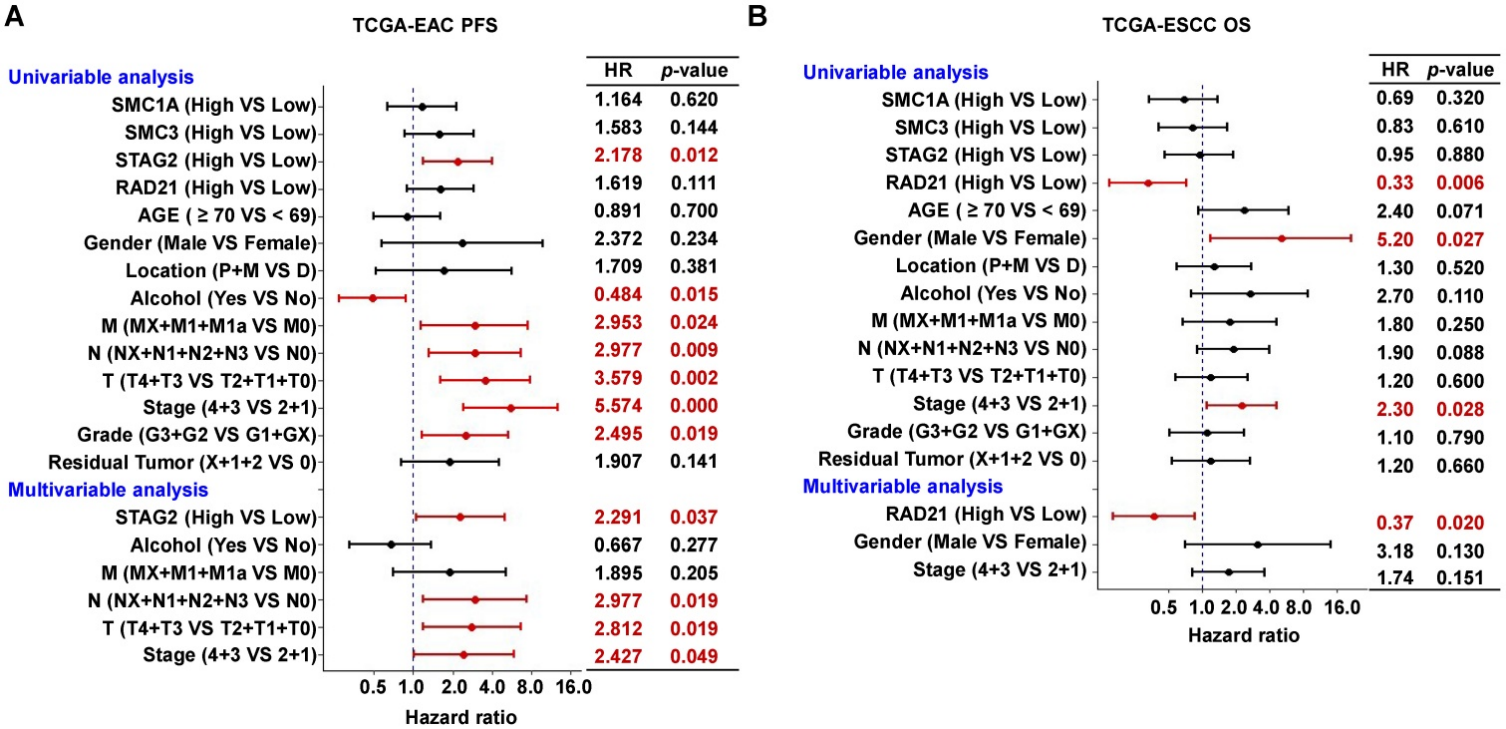
** High STAG2 expression was identified as an independent prognostic risk factor in EAC patients, while high RAD21 expression appears to be an independent prognostic protective factor in ESCC patients.** The forest plot shows the result of univariate and multivariate Cox regression analyses for the associations between **(A)** STAG2 expression and EAC patients' PFS probability and between **(B)** RAD21 expression and ESCC patients' OS probability, respectively, Bars represent the 95% confidence intervals of the hazard ratios. MX represents M stage unknown, NX represents N stage unknown, GX represents grade unknown, and X represents residual tumor unknown.

In univariate Cox regression analysis, we found STAG2, alcohol, M stage, N stage, and clinical stage were significantly associated with OS and PFS of EAC patients (*P* < 0.05; Figure 3A and Supplementary Figure S7A). However, T stage and clinical grade were only significantly associated with PFS in EAC patients (*P* < 0.05; Figure 3A). RAD21, gender, and clinical stage were significantly associated with the OS of ESCC patients (*P* < 0.05; Figure 3B), while only the location was significantly associated with PFS (*P* < 0.05; Supplementary Figure S7B). In multivariate Cox regression analysis, alcohol was an independent prognostic factor for OS in EAC patients (*P* < 0.05; Supplementary Figure S7A), while STAG2, N stage, T stage, and clinical stage were independent prognostic factors for PFS (*P* < 0.05; Figure 3A). RAD21 was an independent prognostic factor for OS in ESCC patients (*P* < 0.05; Figure 3B). Interestingly, STAG2 and RAD21 showed opposing independent prognostic trends between PFS in EAC and OS in ESCC patients.

**Figure 4:**
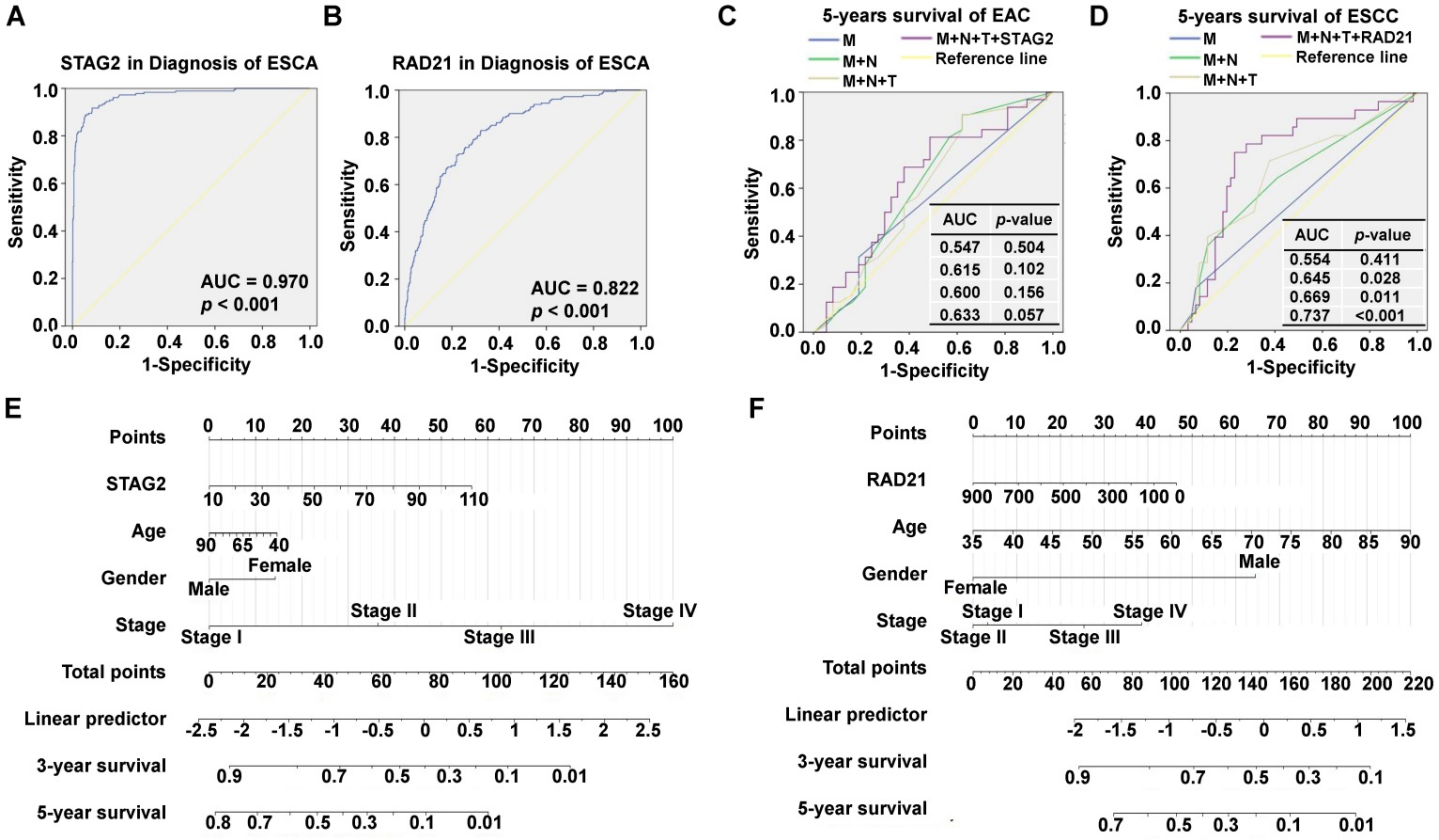
** Diagnostic and prognostic values of STAG2 and RAD21 expression in comparison to other prognostic factors.** ROC analysis of **(A)** STAG2 and **(B)** RAD21 expression in the diagnosis of ESCA. Multiple ROC curves reveal that **(C)** STAG2 expression improved the prognostic accuracy for EAC patients and **(D)** RAD21 expression improved the prognostic accuracy for ESCC patients compared to TNM stage. Nomograms for predicting the OS of **(E)** EAC and **(F)** ESCC patients are shown. Instructions for comprehension of the ROC curves: The x-axis indicates the false-positive rate, which is presented as “1-Specificity.” The y-axis indicates the true-positive rate, which is designated as “Sensitivity.” Instructions for nomogram comprehension: Locate each characteristic on the corresponding variable axis, then draw a vertical line upwards to the points axis to determine the specific point value. Repeat this process. Tally up the total points value and locate it on the total points axis. Draw a vertical line down to the three- or five-year OS to obtain the survival probability for a specific patient.

Furthermore, to investigate the diagnostic ability of STAG2 or RAD21 in ESCA and to compare their predictive value with known clinical prognostic factors, ROC analyses were conducted. The AUC value of STAG2 for diagnosing ESCA was 0.970 (*P* < 0.001; Figure 4A) while RAD21 was 0.822 (*P* < 0.001; Figure 4B). In EAC patients, the AUC value for the five-year survival of the prediction model, including pathological M stage, N stage, T stage, and STAG2 expression, showed a trend of improvement from 0.547 to 0.633 (Figure 4C). In ESCC patients, the AUC value for five-year survival of the prediction model, including pathological M stage, N stage, T stage, and RAD21 expression, was significantly improved from 0.554 to 0.737 (Figure 4D). These results indicated the additive predictive value of STAG2 and RAD21 in EAC and ESCC, respectively, compared to other known prognostic factors. In addition, Nomogram models to predict the three-year and five-year survival of EAC patients (Figure 4E) and ESCC patients (Figure 4F) were developed separately. As shown in the nomogram, the clinical stage contributed the most to the three- and five-year OS, followed closely by the STAG2 expression in EAC patients. The clinical stage also contributed the most to the three- and five-year OS for ESCC patients, followed closely by age and RAD21 expression. These user-friendly graphical tools allowed us to easily determine the three- and five-year OS probabilities for each EAC or ESCC patient.

### Functional and pathway enrichment analyses show that STAG2 promotes EAC development while RAD21 inhibits ESCC progression

**Figure 5:**
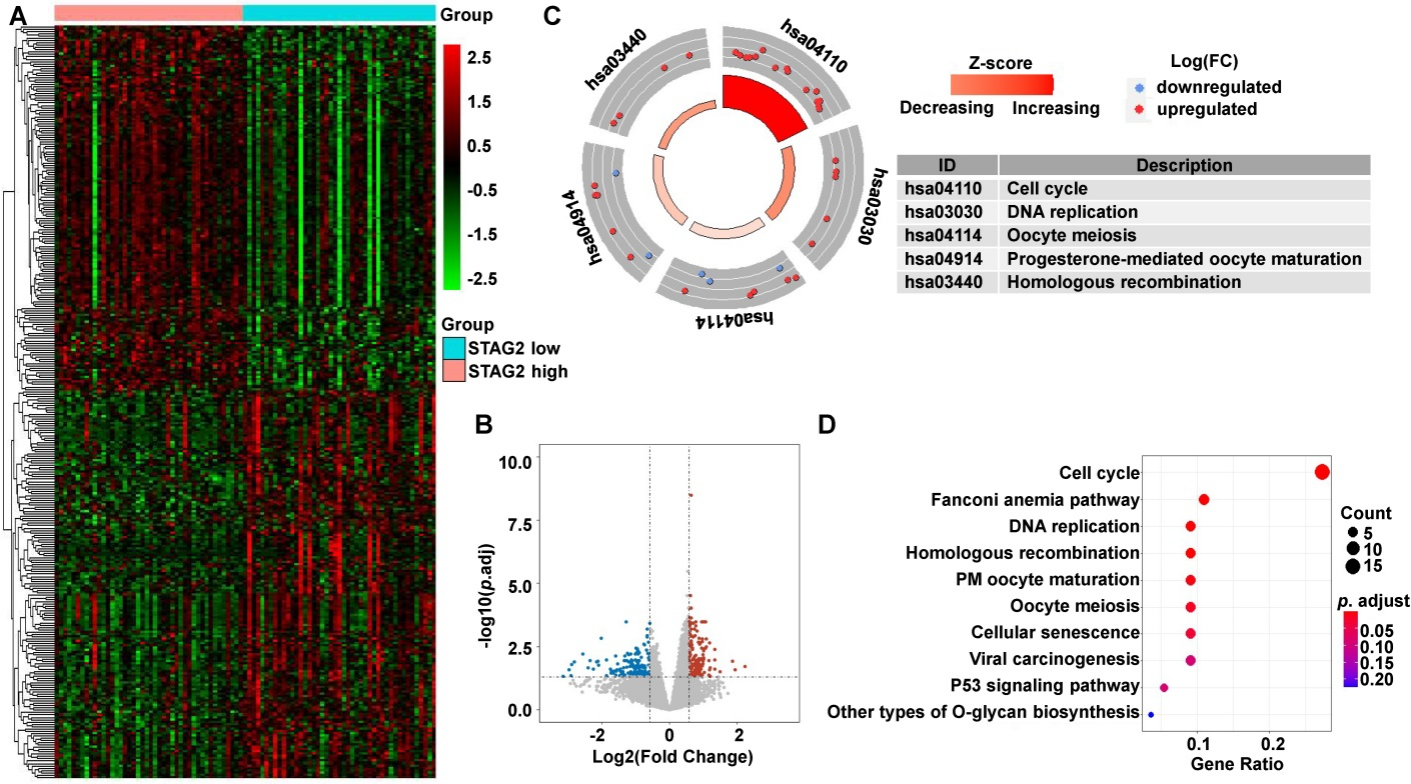
** DEG identification between STAG2 high and low expression EAC patients and pathway enrichment analysis. (A)** Heat map of all the DEGs between STAG2 high with STAG2 low expression groups. Red exhibits overexpression, while blue indicates lower expression of genes in the STAG2 high expression group. **(B)** Volcano plot of differential gene profiles between the STAG2 high and STAG2 low expression groups. Grey nodes represent genes that are not differentially expressed, red nodes represent significantly upregulated genes, and blue nodes indicate significantly downregulated genes in the STAG2 high expression group. **(C)** The circle plot of KEGG enrichment analysis of all significant DEGs. Each spot in the circle represents a gene, and the outer circle refers to significant enrichment signaling pathways IDs. The inner circle shows the Z-score, with the color intensity corresponding to the value of the Z-score. The right table annotates the specific KEGG pathways.** (D)** The bubble plot of KEGG enrichment analysis of all the significantly upregulated DEGs. The x-axis represents the gene ratio, while the y-axis displays the KEGG pathways. The color represents the *P*-value.

To uncover the potential mechanisms associated with STAG2 expression in EAC and RAD21 expression in ESCC, we identified the DEGs between high and low STAG2 expression groups in EAC, as well as the DEGs between high and low RAD21 expression groups in ESCC. In EAC, heatmap and volcano plot analysis identified 326 DEGs. Among them, 158 genes were upregulated and 168 genes were downregulated (Figures 5A and 5B). The biological functions of these DEGs were then explored by the KEGG signaling pathway, GO annotation, and GSEA enrichment analysis. KEGG pathway analysis showed that cell cycle, Fanconi anemia pathway, DNA replication, homologous recombination, progesterone-mediated oocyte maturation, oocyte meiosis, and cellular senescence were the most significantly altered pathways in the STAG2 high expression group (*P-*adjusted < 0.05; Figures 5C and 5D).

**Figure 6:**
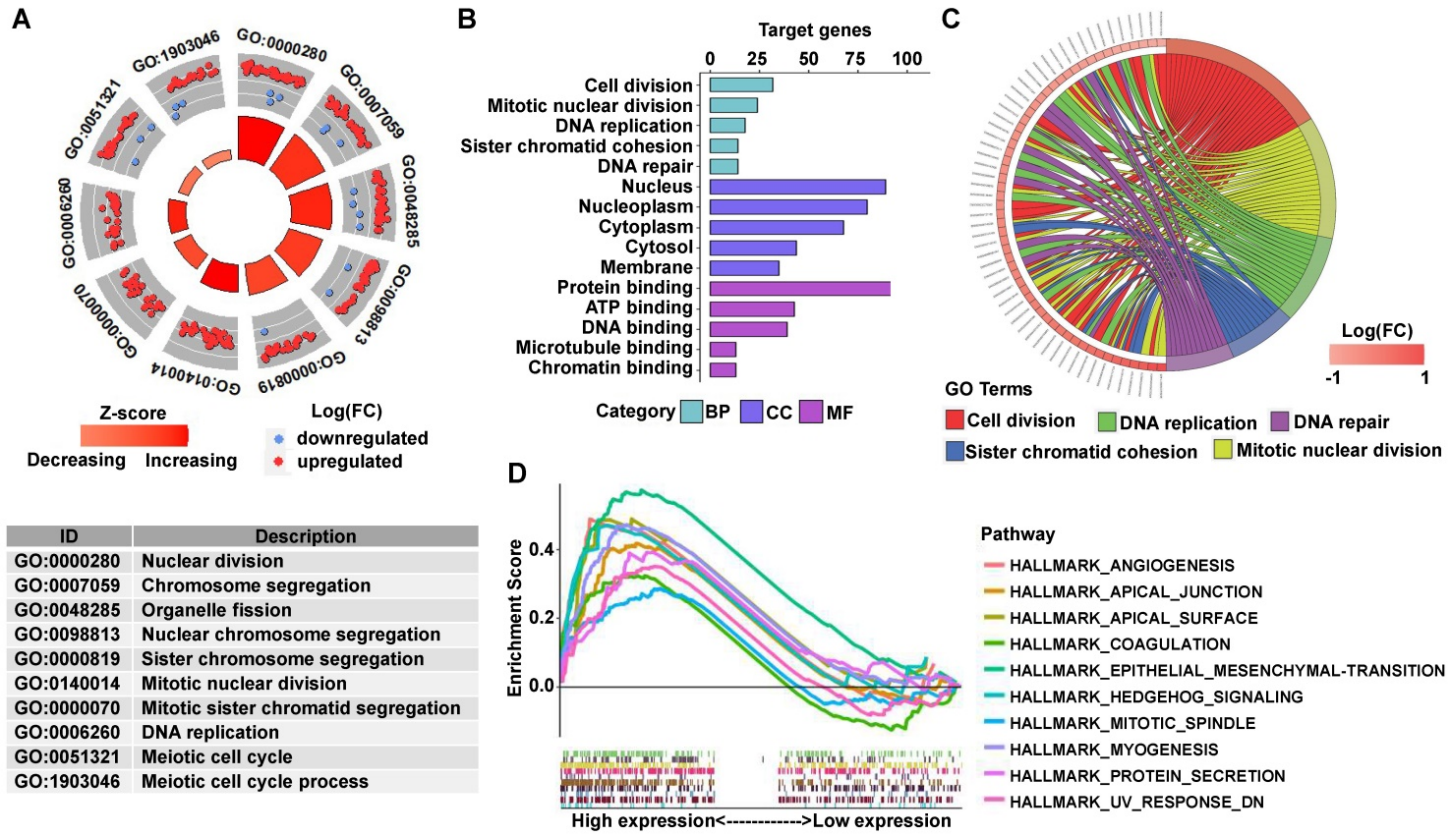
** GO and GSEA enrichment analysis of DEGs between STAG2 high and low expression EAC patients. (A)** The circle plot of GO enrichment analysis of all significant DEGs. Each spot in the circle represents a gene, and the outer circle refers to significant enrichment GO term IDs. The inner circle shows the Z-score, with the color intensity corresponding to the value of the Z-score. The table below annotates the specific GO terms. **(B)** The 15 most significantly enriched GO terms of upregulated mRNAs in the high STAG2 expression group are listed according to their biological processes (BP), cellular component (CC), and molecular functions (MF). The length of each bar indicates the number of enriched genes. **(C)** The chord diagram of significantly enriched GO BP terms in (B). **(D)** The top 10 GSEA enrichment plots of the EAC patients from the TCGA-ESCA dataset between the high and low STAG2 expression groups. A normalized enrichment score of greater than 1 and adjusted *P*-value (false-discovery rate) of less than 0.05 were used to determine significant gene sets.

GO analysis revealed that many biological functions of these DEGs were primarily associated with cell division and DNA repair (Figures 6A-6C). According to the normalized enrichment score of the GSEA enrichment, we selected the most highly enriched signaling pathways. As shown in Figure 6D, the STAG2 high expression group showed genes enriched mainly in aggressive tumor processes, such as angiogenesis, epithelial-mesenchymal transition (EMT), and hedgehog signaling. Furthermore, cell proliferation-related gene sets, including apical junction, apical surface, coagulation, mitotic spindle, and protein secretion, were also significantly enriched in the high STAG2 expression group (Figure 6D).

**Figure 7:**
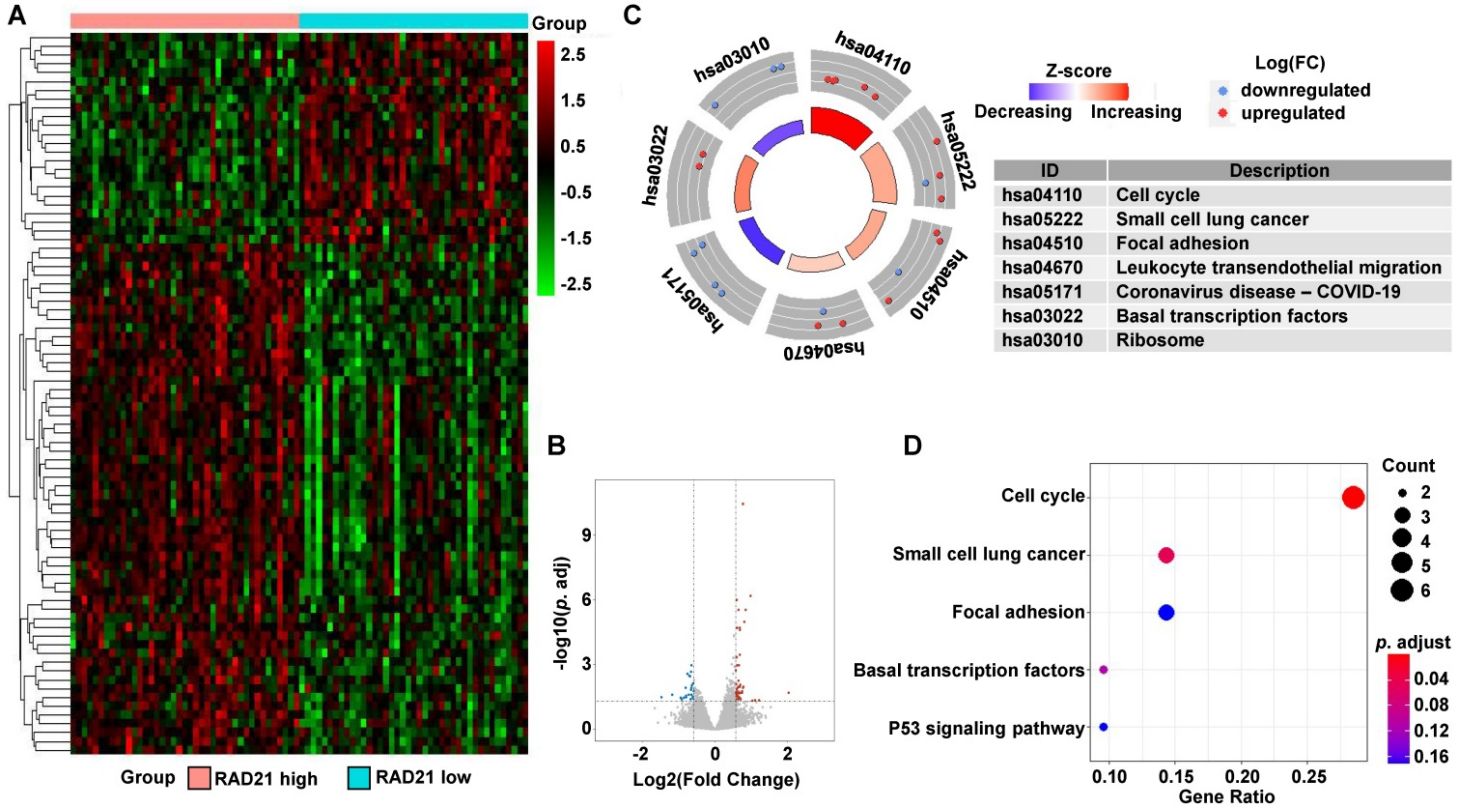
** DEG identification between RAD21 high and low expression ESCC patients and pathway enrichment analysis. (A)** Heat map of all the DEGs between RAD21 high expression with RAD21 low groups. Red exhibits overexpression, while blue indicates lower expression of genes in the RAD21 high expression group. **(B)** Volcano plot of differential gene profiles between the RAD21 high and RAD21 low expression groups. Grey nodes represent genes that are not differentially expressed, red nodes represent significantly upregulated genes, and blue nodes indicate significantly downregulated genes in the RAD21 high expression group. **(C)** The circle plot of KEGG enrichment analysis of all significant DEGs. Each spot in the circle represents a gene, and the outer circle refers to significant enrichment signaling pathways IDs. The inner circle shows the Z-score, with the color intensity corresponding to the value of the Z-score. The right table annotates the specific KEGG pathways. **(D)** The bubble plot of KEGG enrichment analysis of all the significantly upregulated DEGs. The x-axis represents the gene ratio, while the y-axis displays the KEGG pathways. The color represents the *P-*value.

In ESCC, a total of 82 DEGs (58 upregulated and 24 downregulated) were identified between the high and low RAD21 expression groups (Figures 7A and 7B). Biological function analysis of these DEGs was carried out identically with that of EAC above. The KEGG pathway analysis revealed that the most significantly altered pathways in the RAD21 high expression group were cell cycle and small cell lung cancer (*P-*adjusted < 0.05; Figures 7C and 7D).

**Figure 8:**
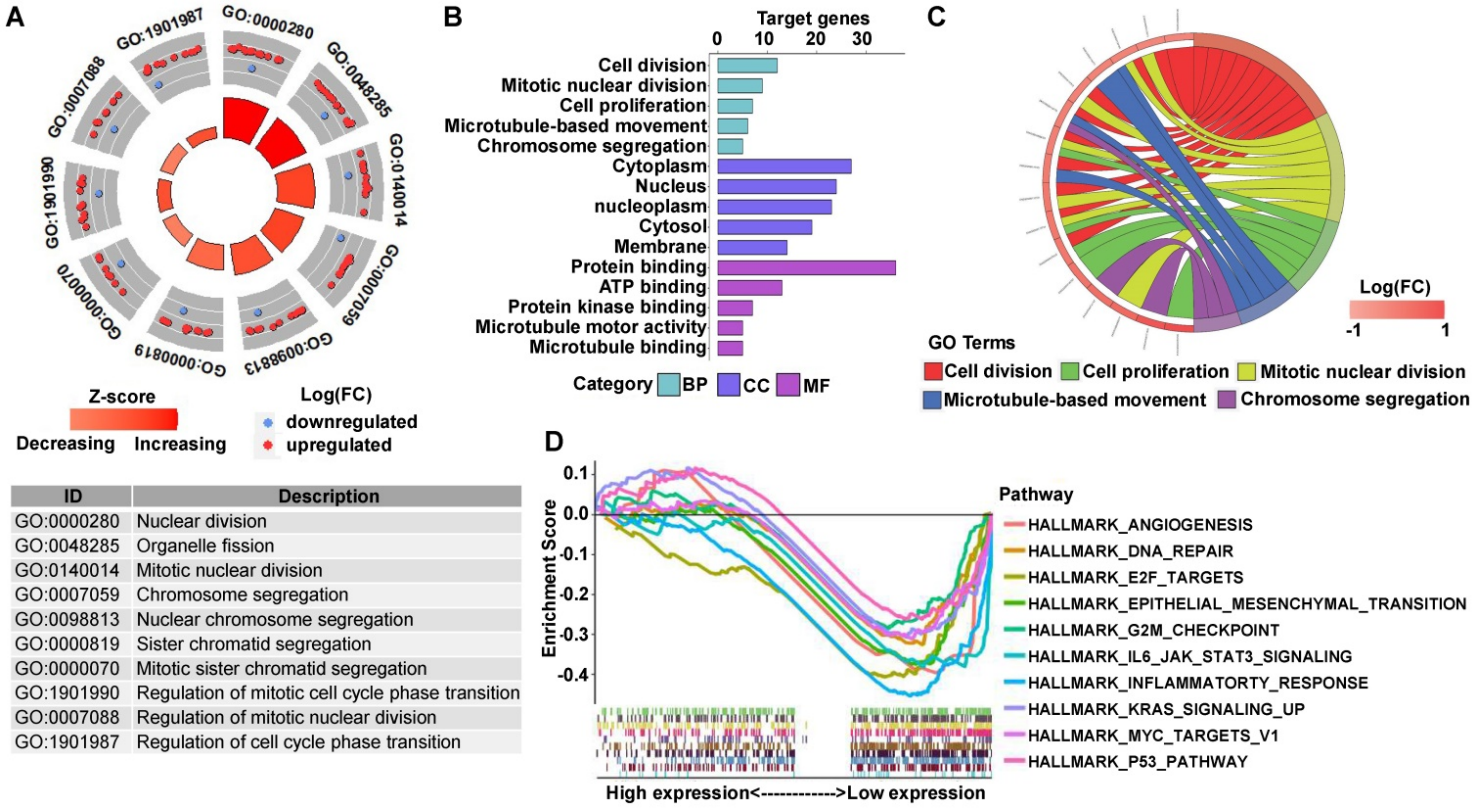
** GO and GSEA enrichment analysis of DEGs between RAD21 high and low expression ESCC patients. (A)** The circle plot of GO enrichment analysis of all significant DEGs. Each spot in the circle represents a gene, and the outer circle refers to significant enrichment GO term identifiers. The inner circle shows the Z-score, with the color intensity corresponding to the value of the Z-score. The table below annotates the specific GO terms. **(B)** The 15 most significantly enriched GO terms of upregulated mRNAs in the high RAD21 expression group are listed according to their biological processes (BP), cellular component (CC), and molecular functions (MF). The length of each bar indicates the number of enriched genes. **(C)** The chord diagram of significantly enriched GO BP terms in (B).** (D)** Top 10 GSEA enrichment plots of the ESCC patients from the TCGA-ESCA dataset between high and low RAD21 expression. A normalized enrichment score of less than -1 and adjusted *P*-value (false-discovery rate) of less than 0.05 were used to determine significant gene sets.

GO analysis showed that many biological functions of these DEGs were primarily associated with cell division (Figures 8A-8C). However, GSEA showed that in the RAD21 low expression group, the DEGs were mainly enriched in genes associated with aggressive tumor processes, such as angiogenesis, E2F targets, EMT, G2/M checkpoint, and Kirsten rat sarcoma viral oncogene (KRAS) signaling of the myelocytomatosis (MYC) viral proto-oncogene targets version 1 and P53 pathway (Figure 8D).

### PPI network with cohesin subunits and protein expression test of STAG2 in EAC and RAD21 in ESCC

**Figure 9:**
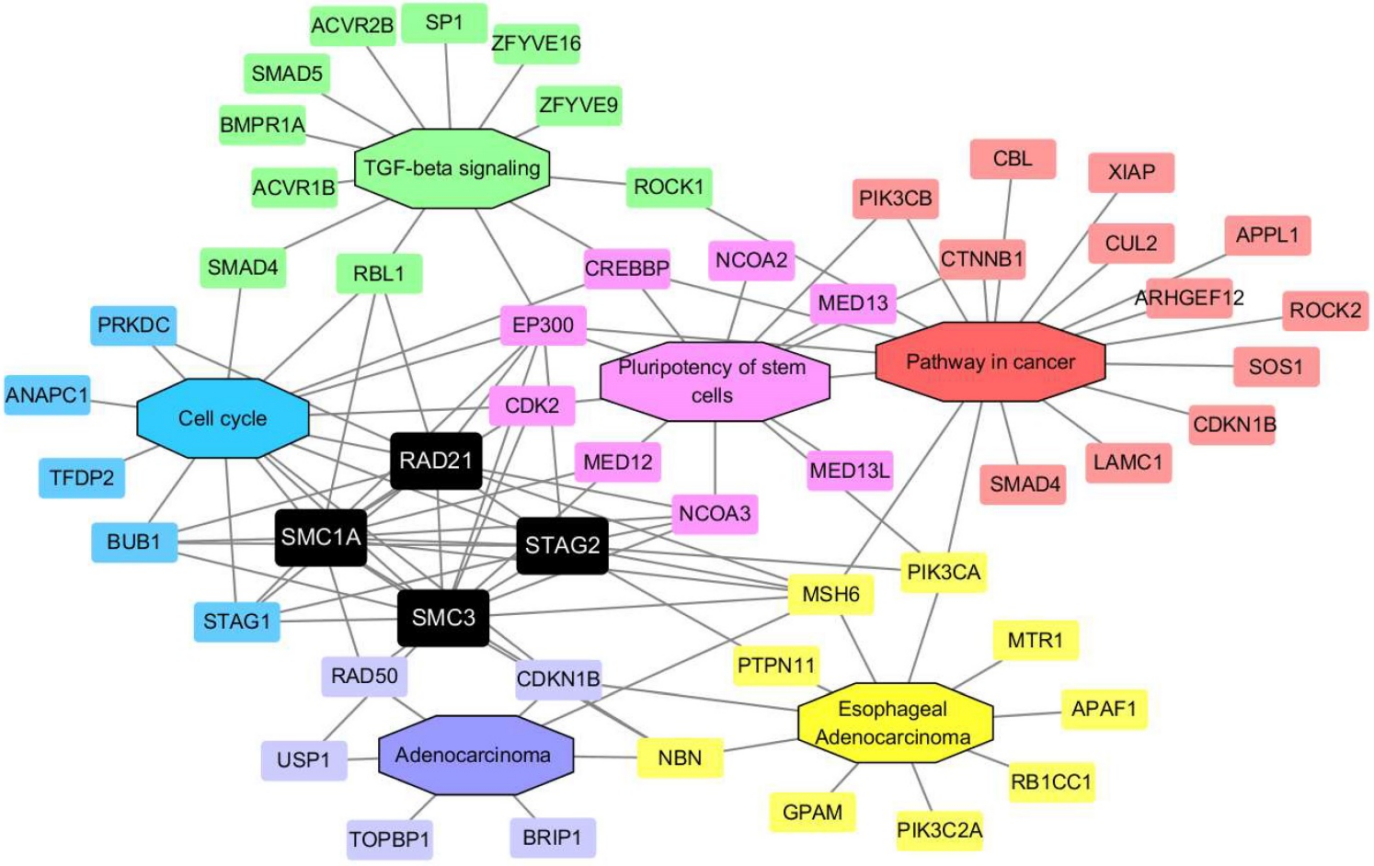
**Cohesin subunit-mediated PPI network construction.** The node color reflects the source of the proteins. Black represents the four subunits of cohesin. Green represents the proteins related with TGF-β signaling. Blue represents cell cycle**-**related proteins. Pink indicates proteins which participate in the pluripotency of stem cells pathways, and red refers to proteins that participate in the pathway in cancer. Purple and yellow denote molecules that participate in adenocarcinoma and EAC, respectively.

To further investigate the possible role of cohesin subunits in ESCA, a PPI network involving 59 proteins was constructed. The protein molecules were separated into the following six groups based on their sources from the DAVID database 29: (I) the proteins exhibited in the TGF-β signaling pathway; (II) the proteins exhibited in the cell cycle pathway; (III) the proteins exhibited in the pluripotency of stem cells pathway; (IV) the proteins exhibited in the pathway in cancer; (V) the proteins exhibited in adenocarcinoma disease; and (VI) and the proteins exhibited in EAC disease. The regulatory network consisted of six modules with different colors, including 59 nodes and 204 edges (Figure 9). As expected, the network demonstrated that STAG2 and RAD21 could cross-talk with other modules, such as TGF-β signaling, pathway in cancer, the pluripotency of stem cells, and adenocarcinoma, in addition to the cell cycle. These may indirectly lead to alterations in tumor development in ESCA.

**Figure 10:**
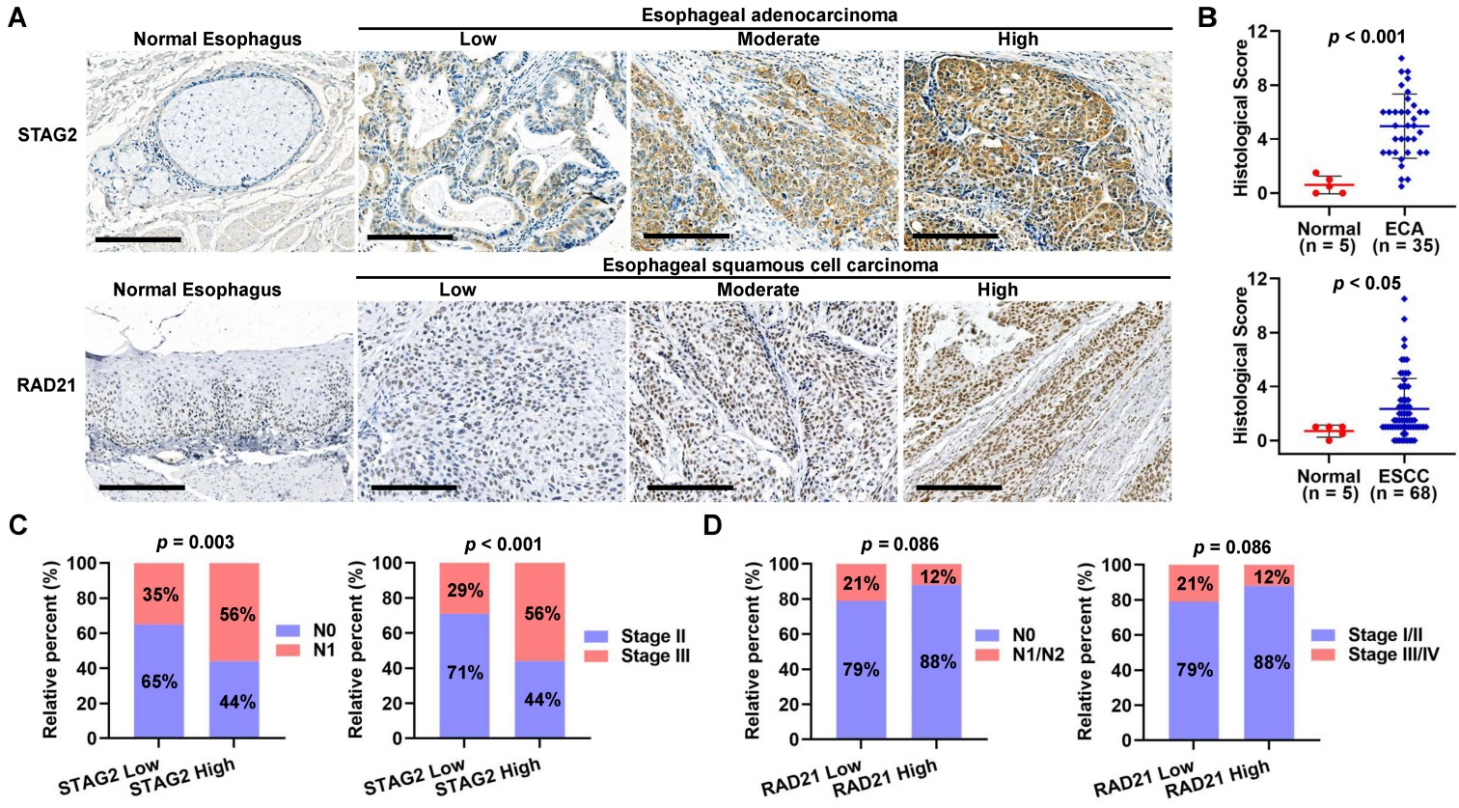
** Protein levels of STAG2 and RAD21 were upregulated in EAC and ESCC tissues, respectively. (A)** Representative micrographs show STAG2 IHC staining of the 35 EAC and five normal esophagus tissue samples and RAD21 IHC staining of 68 ESCC and five normal esophageal tissue samples in the tissue microarray (TMA). Scale bars, 200 µm. **(B)** Quantitative analysis of STAG2 and RAD21 protein expression scores based on the IHC staining of the TMA with EAC or ESCC carcinoma and normal esophageal tissue samples. **(C)** Column charts show the ratio of N0 and N1 stage EAC samples or the ratio of clinical stage II and stage III EAC samples in STAG2 low-HS and high-HS groups. **(D)** Column charts show the ratio of N0 and N1 stage ESCC samples or the ratio of clinical stage II and stage III ESCC samples in RAD21 low-HS and high-HS groups.

To verify the protein expression level of STAG2 and RAD21 in TMA samples of ESCA patients, we performed an IHC analysis. As shown in Figure 10A, STAG2 protein was mainly localized to the nucleus and cytoplasm in the EAC cells, and RAD21 protein was mainly localized to the nucleus in the ESCC cells. HS analysis results showed that STAG2 protein expression levels in EAC cells were significantly higher than in normal esophagus glandular epithelium (Figure 10B). In contrast, RAD21 protein was significantly higher in ESCC cells than in normal esophagus squamous epithelium. A total of 35 EAC samples were divided into two groups by using median HS of STAG2 staining. Chi-square tests revealed that high STAG2 expressed samples significantly correlated with a higher ratio of severe N and clinical stage (Figure 10C). Similarly, 68 ESCC samples were grouped into low- and high-RAD21 expression samples. We found no significant differences in the ratio between the worse N and clinical stages between the two groups. However, high-RAD21 expressing samples tended to show a lower ratio between the severe N and clinical stages (Figure 10D).

## Discussion

EAC originates from glandular epithelial cells and typically develops in the lower third of the esophagus. ESCC primarily develops from the squamous epithelial cells making up the inner lining of the esophagus 30. EAC exhibits features of the chromosomal instability subtype of gastric cancer, whereas ESCC shares molecular similarities with head and neck squamous cell cancer 31. Hence, ESCA should be carefully stratified based on prognosis, risk assessment, and molecular subtypes in clinical treatment.

Cohesin has been demonstrated as an important regulator of cellular stemness and differentiation based on its known role as a chromatin regulator currently 32-36. The regulation and function of cohesin may be tissue-specific, and mutations in cohesin are more prominent in certain types of tumors 37. Recent advances in bladder cancer, colorectal cancer, breast cancer, hepatocellular carcinoma, prostate cancer, and Ewing sarcoma studies have demonstrated that cohesin subunits play a pivotal role in the genesis and development of human tumors 38-43. However, few studies have focused on the significance of cohesin subunits in ESCA. Data mining strategies using publicly accessible databases and integrative bioinformatics analysis have become a powerful method for retrospective cancer research in recent years 44-46. Therefore, the present study aimed to reveal the cohesin-related molecular mechanism associated with the pathogenesis of different subtypes of ESCA using publicly available datasets and comprehensive bioinformatics approaches.

In the present study, we investigated the clinical significance of cohesin subunits and direct regulators by analyzing the RNA-seq data from TCGA, ESCA, and GTEx datasets. Our study showed that mRNA levels of all cohesin subunits and direct regulators were significantly upregulated in the ESCA tissues compared to normal esophageal tissues. Among 12 analyzed genes, the expression levels of STAG2 and SMC1B were associated with the OS of ESCA patients. However, the mRNA levels of SMC1B were extremely low in all TCGA-ESCA samples and showed no significant differences in the following stratification analysis of EAC or ESCC. This is most likely due to SMC1B only exhibiting in meiotic cohesin but not mitotic cohesin 47. Interestingly, STAG2 and RAD21 emerged with distinct differences in the following stratification analysis of ESCA. In subtype EAC, OS and PFS of EAC patients with high STAG2 expression were significantly shorter than those with low STAG2 expression. Thus, STAG2 was identified as an independent risk prognostic factor for EAC patients, suggesting that STAG2 can be used as a predictive biomarker for risk assessment and prognosis in EAC. STAG1 is a STAG2 homolog with distinct functions in cohesin biology 36, 48. Several studies have proved that STAG1 inactivation imparts a potent synthetic lethality in STAG2-mutant cancer cells 49-51. However, STAG1 expression in the present study showed no significant correlation with the survival status of EAC or ESCC patients, although STAG1 showed a similar trend to that of STAG2. STAG2 expression could improve the accuracy of the five-year survival prediction model built by pathological M stage, N stage, and T stage. The expression level of STAG2 also acts as an important risk predictor for the three-year and five-year survival of EAC patients. By comparison, in the ESCC subtype, the OS of patients with high RAD21 expression was significantly longer than those with low RAD21 expression. Thus, RAD21 was unexpectedly identified to be an independent protective factor of OS for ESCC patients. RAD21 could also improve the accuracy rate of the five-year survival prediction model, and lower RAD21 expression acts as an important risk predictor for the three-year and five-year survival of EAC patients. Using TMA, STAG2 protein expression was positively correlated with lymph node metastasis and advanced clinical stage of EAC patients, whereas there appeared to be a negative correlation between RAD21 protein expression and the malignant clinicopathological parameters in ESCC. These results are consistent with some other tumor studies in the literature. For example, some studies supported that lower STAG2 expression is beneficial for bladder cancer patient outcomes 52-54, while other researchers directly contradict this point 38, 55. Overexpression of RAD21 was linked with poor disease outcome and resistance to chemotherapy in breast cancer 21, 56, while low RAD21 expression characterized metastases in oral squamous cell carcinoma 23. Thus, these conflicting findings indicate that discrepancies between STAG2 and RAD21 in different ESCA subtypes in the present study may derive from the underlying mechanisms pertinent to specific biological properties in cancer cells. Exploring the mechanisms of STAG2 in EAC and RAD21 in ESCC may help develop novel therapeutic approaches for ESCA patients.

Biological pathway analysis and functional enrichment analysis in this study illustrated that cell division-related processes, tumor vascularization, EMT, and cancer signaling pathways, such as hedgehog signaling, were significantly enriched in the high STAG2 expression group in EAC patients. However, in ESCC patients, the angiogenesis pathway, EMT, and cell proliferation-related pathways, such as E2F/MYC targets, and tumorigenesis-related pathways, such as KRAS signaling and P53 pathways, were all significantly enriched in the low RAD21 expression group. Angiogenesis and EMT have been known to be essential for the growth and metastasis in many solid tumors in the last few decades 52-54. However, STAG2 and RAD21 have not been reported to affect tumor metastasis directly. In the present study, we discovered overexpressed STAG2 might promote EAC progression and metastasis through facilitating tumor vascularization and EMT. A relatively higher level of RAD21 tends to inhibit ESCC progression and metastasis compared to a lower level; however, the underlying specific mechanisms of these conflicting actions still need further investigation.

The possible explanation for our findings related to the emerging role of cohesin is that the expression levels of cohesin complex and regulator genes were all upregulated due to mitotic activation in tumor cells. Cohesin components also play important roles in some other processes, such as in regulating genomic organization; transcription; and controlling cellular differentiation by generating, maintaining, and regulating the intra-chromosomal DNA looping events that modulate three-dimensional genome organization 14, 36. Furthermore, cohesin components might exhibit diverse roles in different cancer types or subtypes. For example, low STAG2 expression in muscle-invasive bladder cancer patients had been demonstrated to be associated with less progression compared to high STAG2 expression 57. The protein expression of STAG2 was reported as a prognostic biomarker in low-grade, non-muscle-invasive bladder cancer 38. On the contrary, loss of STAG2 promotes migratory and metastatic potential of Ewing sarcoma cells 58. However, the exact mechanism by which STAG2 drives or suppresses cancer pathogenesis remains unknown.

Another possible cause of tumor progression influenced by cohesin subunits may be the connections between cohesin genes with extracellular matrix (ECM) production 18, 59. It was reported that decreased expression in RAD21 in mesenchymal cancer cells could cause transcriptional activation of TGF-β1 and integrin subunit alpha 5 due to the alteration of intrachromosomal chromatin interactions within their loci 60. TGF-β is extensively implicated in the expression of ECM proteins 61, while dysregulation of ECM can contribute to neoplastic progression 62. This indicates RAD21 may have similar functions in ESCC. Although the PPI network analysis in the present study did not explore the direct interactions between RAD21 and TGFB1 or integrin subunit alpha 5, we discovered that RAD21 could interact with TGF-β signaling cascade through other proteins such as E1A binding protein p300 (EP300) and RB transcriptional co-repressor-like 1).

Further, STAG2 and RAD21 interacted with pluripotency of stem cell signaling pathway-related proteins such as EP300 and nuclear receptor co-activator 3. Moreover, among the interaction network of cohesin subunits, many proteins are known to be involved in cancer or adenocarcinoma pathways, such as mutS homolog 6 and phosphatidylinositol-4,5-bisphosphate 3-kinase catalytic subunit alpha. These findings thus suggest that more attention should be paid to uncovering key proteins in the indirect regulation between cohesin subunits and EMT or cancer-related genes.

There are some limitations to our study that need to be noted. First, only transcriptomic expression of cohesin subunit genes with clinical data was analyzed to predict ESCA prognosis from TCGA databases. Thus, the data were limited. Proteomic analysis should be complementary, and the results should be validated in additional, larger sample sizes. Second, the present research was a retrospective study with selection biases inherent in the cohorts; thus, a prospective study is also needed. Third, although a series of functional annotations and enrichment analyses has been investigated, the molecular mechanisms of STAG2 in EAC and RAD21 in ESCC remain unclear. Further research is required to uncover the potential biological mechanisms of cohesin subunits by using different experimental approaches.

The present study explored the clinical value and biological processes of cohesin subunits using ESCA data in the TCGA database and samples from TMA. To some extent, STAG2 and RAD21 can be used as the prognostic biomarkers for risk assessment and prognostic stratification in ESCA. This study provides potential novel insights into further investigation of ESCA.

## Supplementary Material

Supplementary figures.

## Abbreviations

ESCA: esophageal carcinoma
OS: overall survival
PFS: progression-free survival
EAC: esophageal adenocarcinoma
ESCC: esophageal squamous cell carcinoma
EMT: epithelial-mesenchymal transition
PPI: protein-protein interaction
TMA: tissue microarray
SMC: structural maintenance of chromosomes
GTEx: Genotype-Tissue Expression
IHC: immunohistochemical/immunohistochemistry
TCGA: The Cancer Genome Atlas
ROC: receiver-operating characteristic curve
AUC: area under the receiver-operating characteristic curve
DEGs: differentially expressed genes
KEGG: Kyoto Encyclopedia of Genes and Genomes
GO: Gene Ontology
GSEA: gene set enrichment analysis
FDR: false-discovery rate
BP: biological processes
MF: molecular function
CC: cellular component
STRING: Search Tool for the Retrieval of Interacting Genes
DAVID: Database for Annotation, Visualization, and Integrated Discovery
HS: histological score
