# Supplementary material for: Prognostic Values and Underlying Regulatory Network of Cohesin Subunits in Esophageal Carcinoma
Source: J Cancer. 2022 Mar 6;13(5):1588–602. doi: 10.7150/jca.66949 (PMC8965109; doi:10.7150/jca.66949)
Supplement: Supplementary file 1 — Supplementary figures. [file jcav13p1588s1.pdf]

A

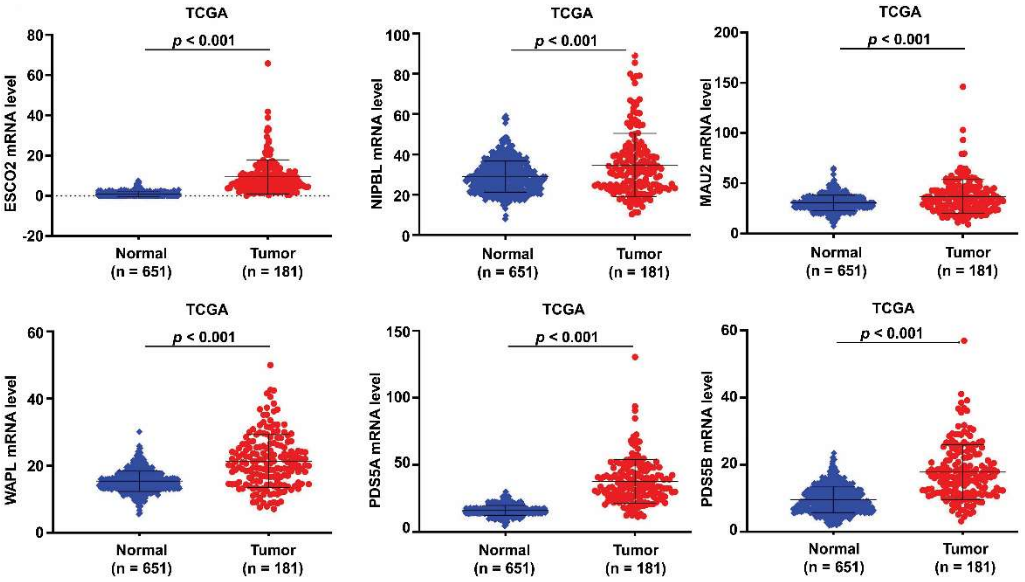

B

Analysis Type by Cancer	Cancer vs. Normal											
	ESCO2		NIPBL		MAU2		WAPL		PDS5A		PDS5B	
Bladder Cancer	2	3	6	4	4	4	3	5	3	3		4
Brain and CNS Cancer	11	1	12	7	15	11	10	11	16	4	10	21
Breast Cancer	25	4	23	19	10	12	14	16	14	14	10	30
Cervical Cancer	2	1	7	1	1	5	2	5	2	7	4	7
Colorectal Cancer	23	14	21	9	24	2	16	18	18	9	29	10
Esophageal Cancer	2	1	5	4		7	4	5	1	5	6	2
Gastric Cancer	6		15		5	3	8	2	6	9	16	1
Head and Neck Cancer	4	4	14	7	2	11	12	12	15	3	10	15
Kidney Cancer	2	3	13	2	7	8	11	4	12	5	7	7
Leukemia	4	10	16	10	13	15	8	14	17	9	10	10
Liver Cancer	1	5	7	3	5	5	4	4	4	5	4	8
Lung Cancer	9	8	14	5	7	10	2	20	12	8	3	19
Lymphoma	18	6	8	17	9	14	5	12	5	22	11	19
Melanoma		1	3	3	2		1	3	1	1	4	3
Myeloma		3	5		4	1	3	4	4	2	2	4
Other Cancer	6	2	14	8	14	11	12	7	16	9	7	17
Ovarian Cancer	4	1	7	5	6	3	2	3	8	6	4	11
Pancreatic Cancer	1	2	6	1	2	6	5	1	9		4	3
Prostate Cancer	2	3	5	3	8	4	2	5	8	1	1	8
Sarcoma		1	15	3	12	7	6	11	13	2	10	2
**Significant Unique**	122	73	214	109	147	138	129	160	181	123	152	201
**Total Unique Analyses**	301		394		394		407		433		433	

1 5 10 10 5 1

%

Figure S1. The mRNA levels of cohesin direct regulators are upregulated in the ESCA tissues and are altered in different human cancers. (A) The relative mRNA expression levels of cohesin regulators in normal esophagus and ESCA tissues from the GTEx and TCGA-ESCA datasets. (B) Oncomine database analysis results of cohesin regulator mRNA levels in the tumor and normal tissues in human cancers. Note: Red and blue denote upregulation and downregulation of the genes of cohesin regulators in the tumor tissues, respectively.

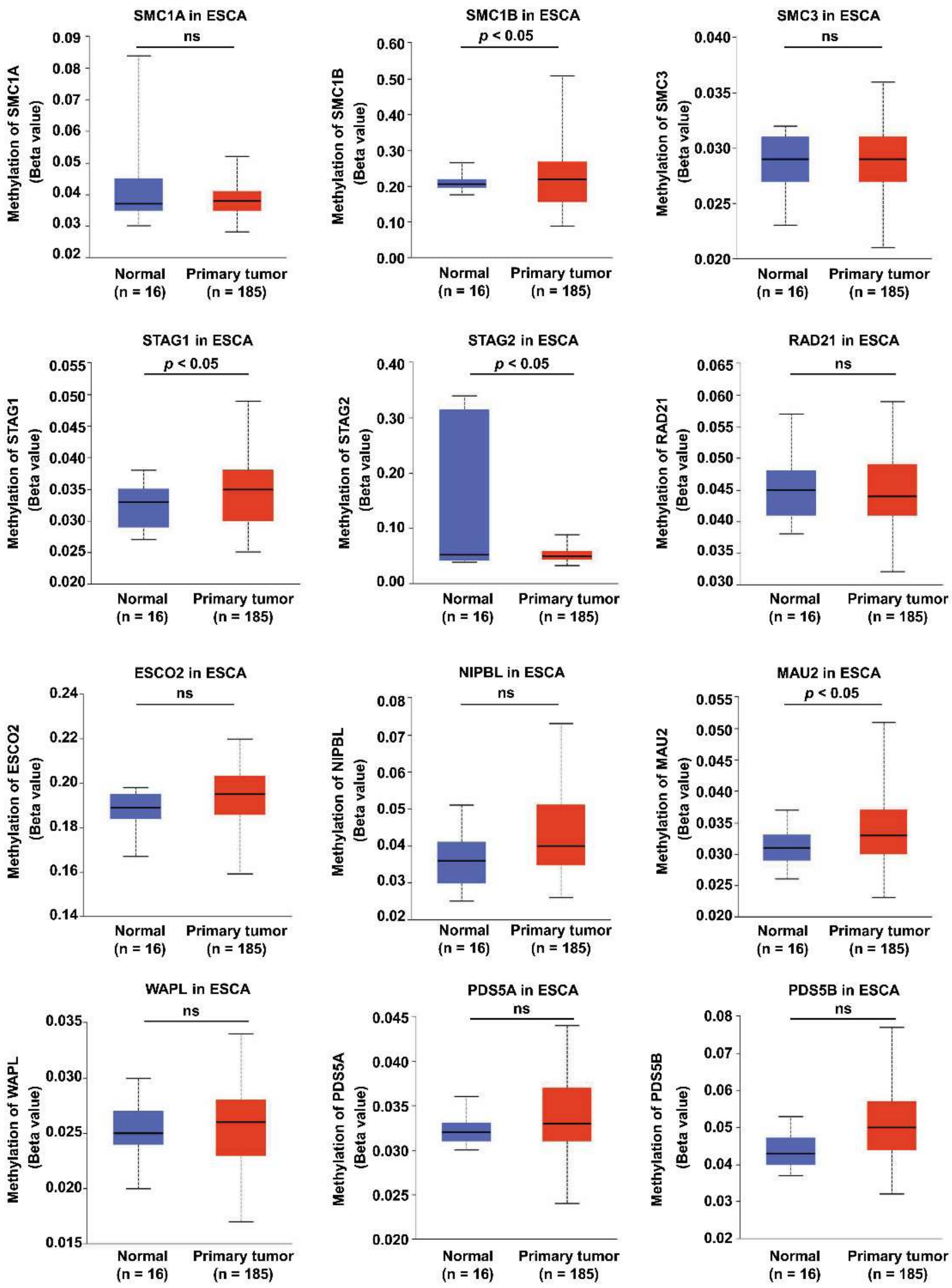

Figure S2 DNA methylation analysis of cohesin subunit and regulator genes in the ESCA patients. Boxplots show the methylation level of the CpG island of cohesin subunit and regulator genes between ESCA tissues and normal esophageal tissue samples.

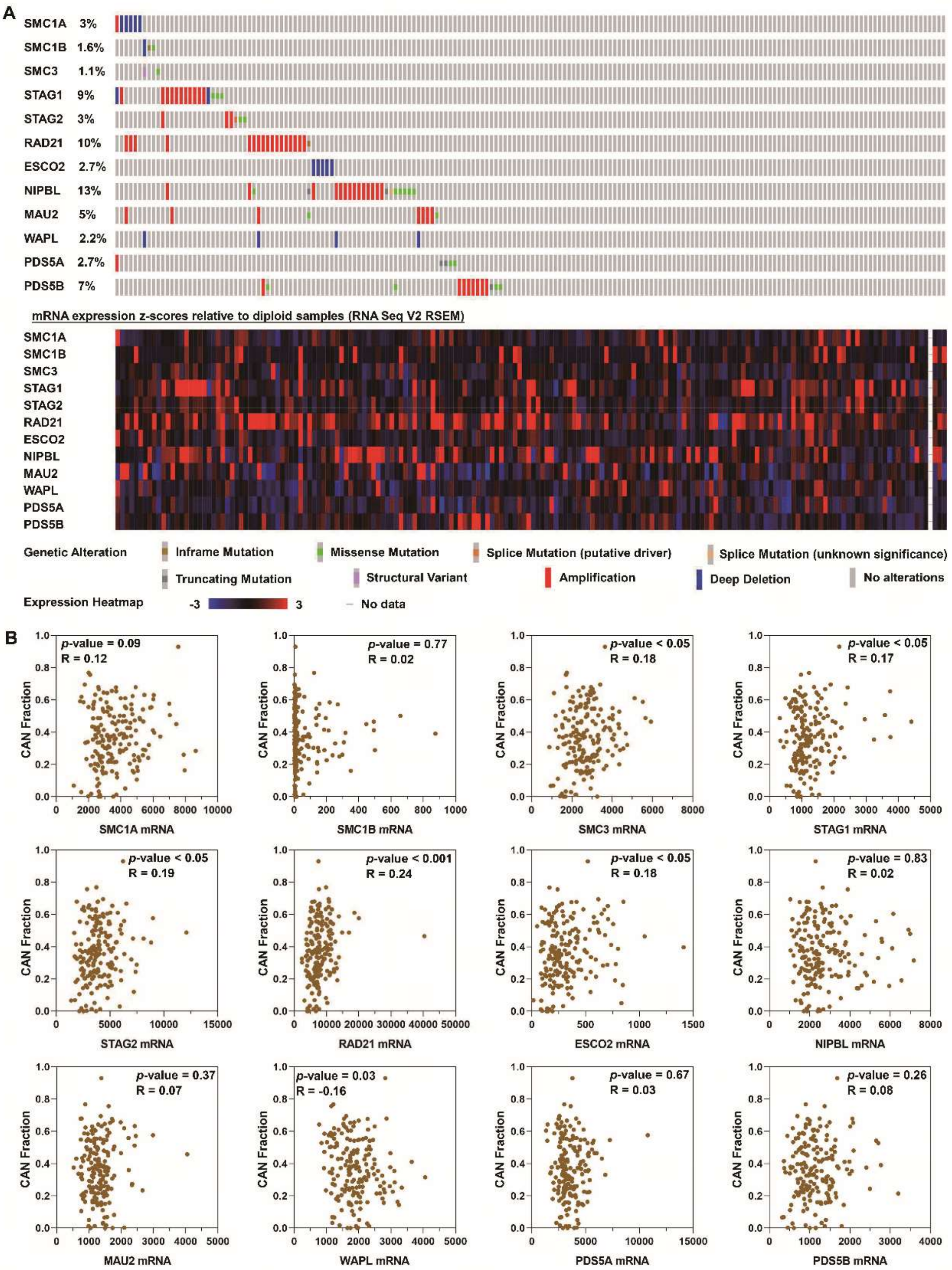

Figure S3 Gene alterations have little correlation with the mRNA expression of cohesin subunits and regulators in ESCA patients. (A) Gene alteration status and the mRNA expression heatmap of cohesin subunit and regulator genes of TCGA ESCA patients from the cBioPortal database. (B) Scatter plots show Spearman's correlation between copy number alteration (CNA) and the expression levels of cohesin subunit and regulator genes in the ESCA samples. R denotes Spearman's correlation coefficient.

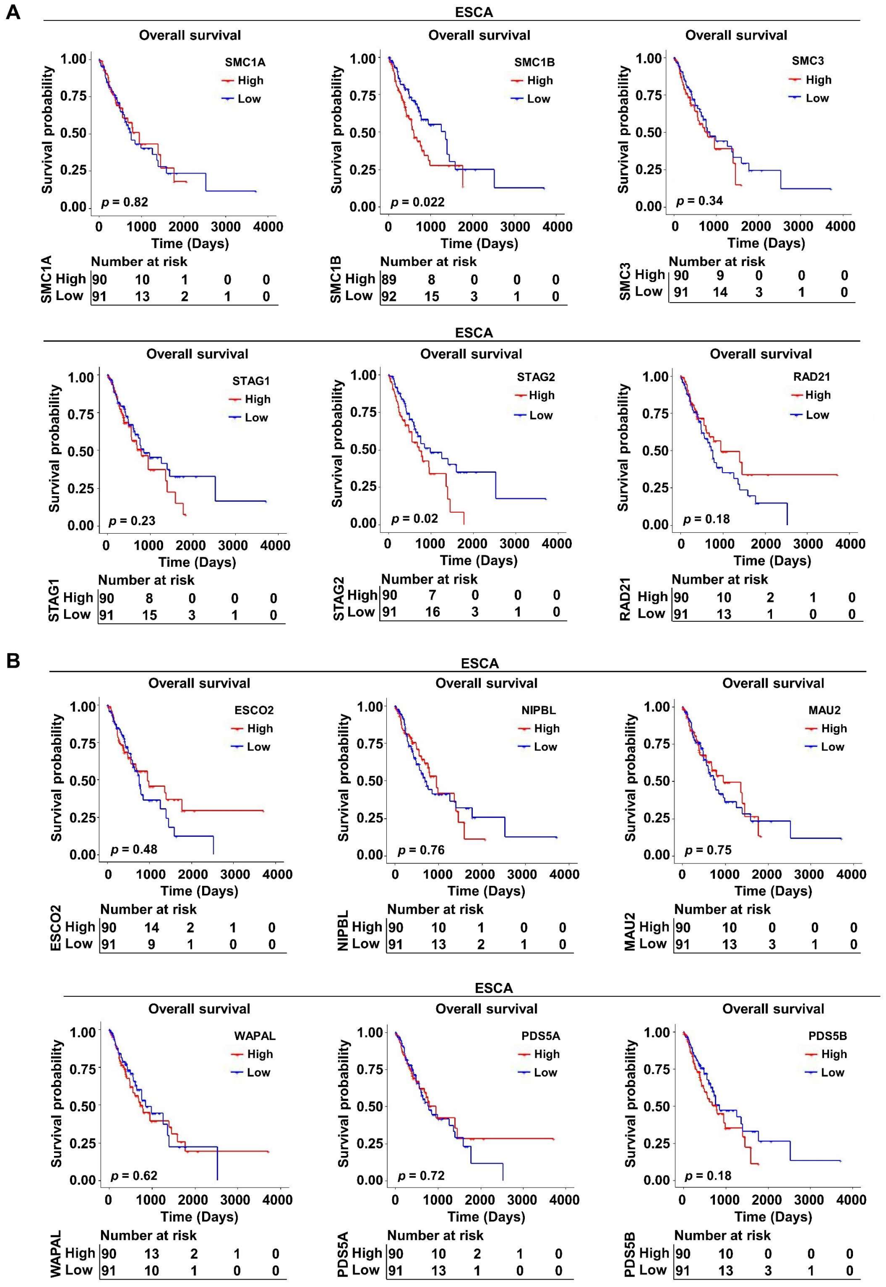

Figure S4 High STAG2 and SMC1B expression correlate with poor survival outcomes among cohesin subunit and regulator genes in ESCA patients. Kaplan-Meier survival curves show OS of the ESCA patients with high- and low-expression of (A) cohesin subunit genes and (B) cohesin regulator genes from TCGA database.

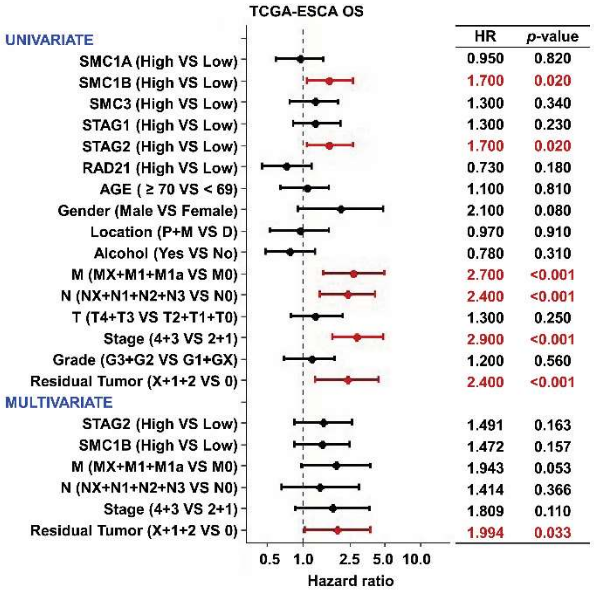

Figure S5 STAG2 and SMC1B expression levels associate with OS of ESCA patients but are not independent prognostic factors. Forest plot shows the result of univariate and multivariate Cox regression analysis for the association between the expression of cohesin subunits and OS probability in ESCA patients. MX represents M stage unknown, NX represents N stage unknown, GX represents grade unknown, and X represents residual tumor unknown.

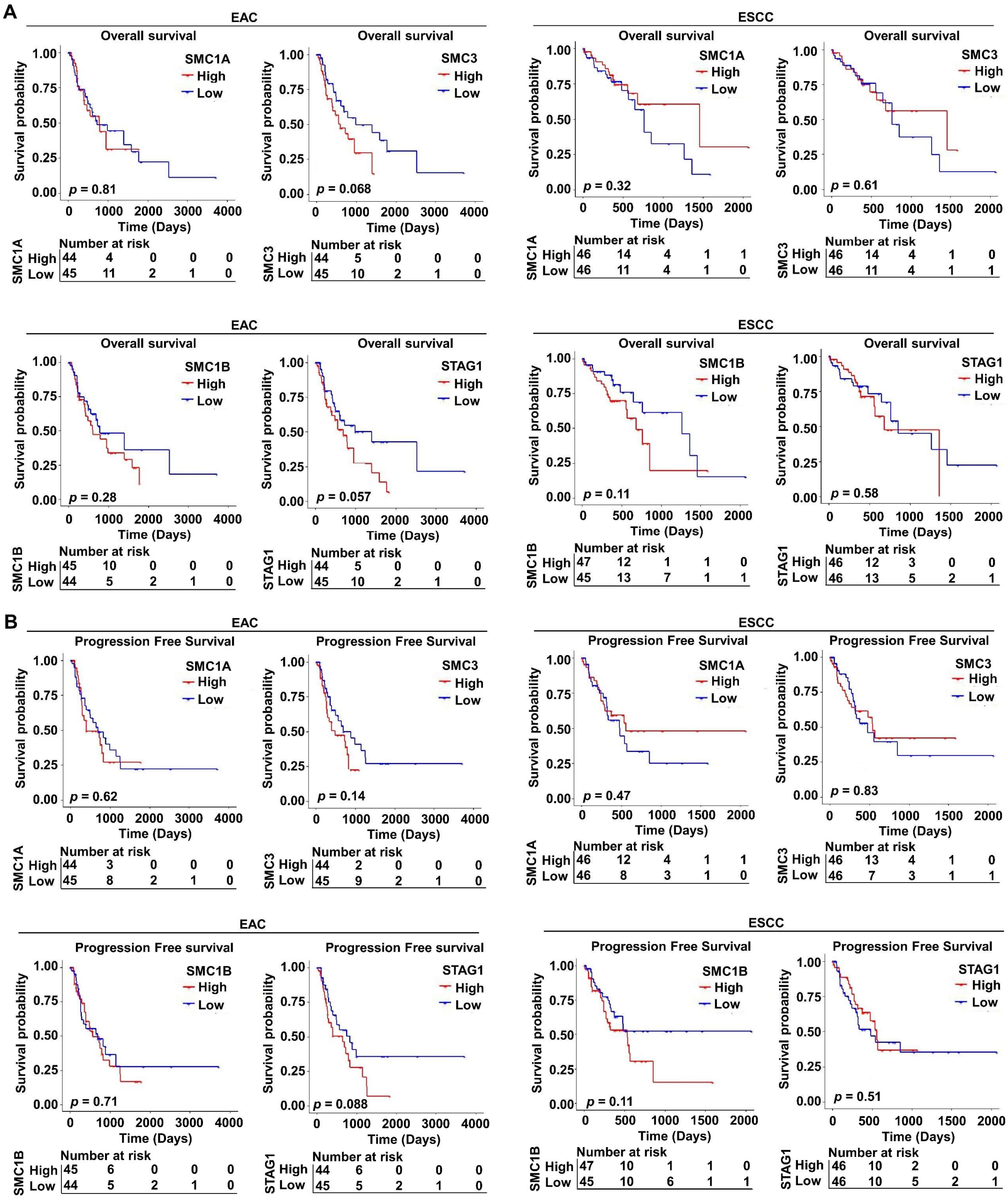

Figure S6 SMC1A, SMC1B, SMC3 or STAG1 expression is uncorrelated with survival outcomes among cohesin subunit genes in EAC or ESCC patients. Kaplan-Meier survival curves show (A) OS and (B) PFS of the EAC or ESCC patients with high- and low-expression of SMC1A, SMC1B, SMC3 and STAG1 from the TCGA database.

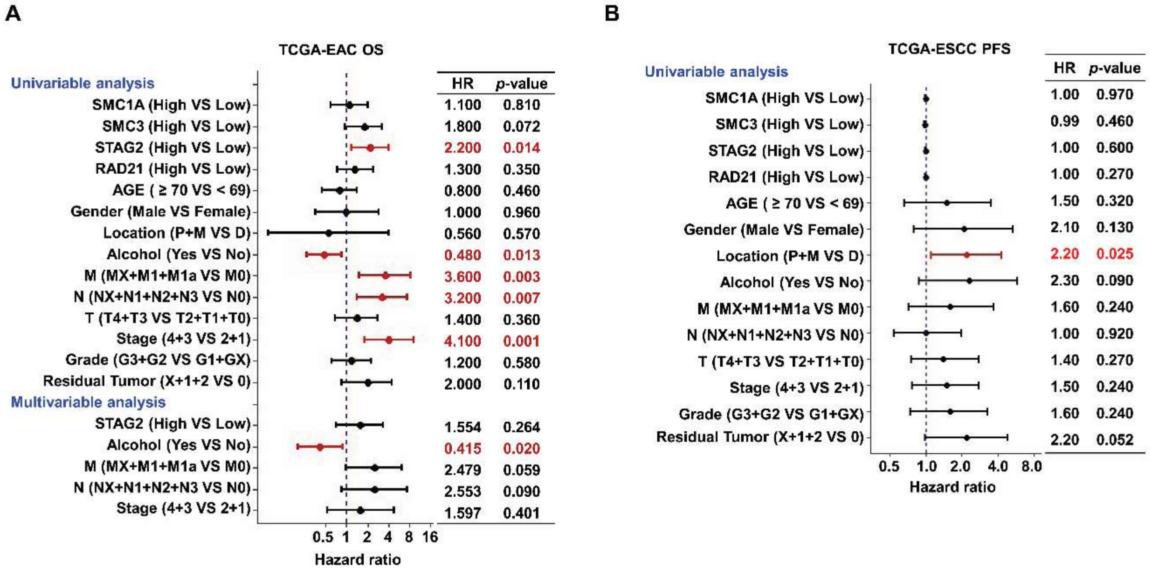

Figure S7 STAG2 expression is not an independent OS prognostic risk factor in EAC patients, while RAD21 expression shows no significance in hazard regression in PFS of ESCC patients. Forest plot shows the result of univariate and multivariate Cox regression analysis for the association between (A) STAG2 expression and EAC patient OS probability and between (B) RAD21 expression and ESCC patient PFS probability. Bars represent the 95% confidence intervals of the hazard ratio. MX represents M stage unknown, NX represents N stage unknown, GX represents grade unknown, and X represents residual tumor unknown.
